# Do male engagement and couples’ communication influence maternal health care-seeking? Findings from a household survey in Mozambique

**DOI:** 10.1186/s12884-020-02984-y

**Published:** 2020-06-11

**Authors:** Gilda G. Sitefane, Joya Banerjee, Diwakar Mohan, Connie S. Lee, Jim Ricca, Myra L. Betron, Rosa Marlene Manjate Cuco

**Affiliations:** 1 Maternal and Child Survival Program/ Jhpiego, Avenida Armando Tivane n°, 1620 Maputo, Mozambique; 2 Maternal and Child Survival Program/ Jhpiego, 1776 Massachusetts Ave, NW, Suite 300, Washington, DC 20036 USA; 3 The Johns Hopkins University Bloomberg School of Public Health, 615 N. Wolfe Street, Baltimore, MD 21205 USA; 4 Faculty of Medicine of Eduardo Mondlane University and National Ministry of Health, Eduardo Mondlane Ave, Maputo, Mozambique

**Keywords:** Gender, Male engagement, Couples’ communication, Birth preparedness, Complication readiness, Institutional delivery, Mozambique, Maternal health, Family planning, Antenatal care

## Abstract

**Background:**

This study explored effects of couples’ communication and male participation in birth preparedness and complication readiness (BPCR) on delivery in a health facility (“institutional delivery”). A cross-sectional, baseline household survey was conducted in November 2016 prior to an integrated maternal and child health project in Nampula and Sofala Provinces in Mozambique.

**Methods:**

The study used the Knowledge, Practices and Coverage survey tool, a condensed version of the Demographic and Health Survey and other tools. The sample included 1422 women. Multivariable logit regression models tested the association of institutional delivery with couples’ communication and four elements of BPCR both with and without male partners: 1) saving money, 2) arranging transport, 3) choosing a birth companion, and 4) choosing a delivery site; controlling for partners’ attendance in antenatal care and social and demographic determinants (education, wealth, urban/rural location, and province).

**Results:**

The odds that women would deliver in a health facility were 46% greater (adjusted odds ratio (aOR) = 1.46, 95% confidence interval (CI) = 1.02–2.10, *p* = 0.04) amongst women who discussed family planning with their partners than those who did not. Approximately half of this effect was mediated through BPCR. When a woman arranged transport on her own, there was no significant increase in institutional delivery, but with partner involvement, there was a larger, significant association (aOR = 4.31, 2.64–7.02). Similarly, when a woman chose a delivery site on her own, there was no significant association with institutional delivery (aOR 1.52,0.81–2.83), but with her partner, there was a larger and significant association (aOR 1.98, 1.16–3.36). Neither saving money nor choosing a birth companion showed a significant association with institutional delivery—with or without partner involvement. The odds of delivering in a facility were 28% less amongst poor women whose partners did not participate in BPCR than wealthy women, but when partners helped choose a place of delivery and arrange transport, this gap was nearly eliminated.

**Conclusions:**

Our findings add to growing global evidence that men play an important role in improving maternal and newborn health, particularly through BPCR, and that couples’ communication is a key approach for promoting high-impact health behaviors.

## Background

Mozambique’s maternal mortality ratio stands at 489 per 100,000 live births as of 2015, one of the highest in the world [1]. The country has made significant progress in encouraging women to deliver in health facilities, with a nationwide institutional delivery rate of 70.3% [2]. However, progress has been uneven, with a rate of 88% in Sofala Province but only 68% in Nampula [2]. A scoping study in three provinces in Mozambique found that the most significant barriers to giving birth in a health facility and delays in seeking care were mostly due to gender barriers, particularly the influence of family and partners [3].

In Mozambique, women are seldom the main decision makers in relationships [4], they have lower levels of literacy than men, [5] and gender and social norms often dictate that they obey their male partners [6]. A study by Audet et al. on the barriers to male involvement in antenatal care (ANC) in Zambézia Province of Mozambique, found that women had the lowest proportion of ANC uptake in the country, despite the availability of free services. The authors identified gender inequality and a lack of male support as one of the main causes of low ANC participation, and noted that “[a]ll groups participating in this study discussed the need for male partners to provide logistic, financial, and psychological support to increase uptake of ANC services.” [7] Women were considered responsible for the health of the baby during pregnancy, and men did not see the baby as their responsibility until after birth [7]. In some cases, men did want to be involved, but there was a lack of support for male involvement from members of their community, particularly from other men. Men who provided emotional or physical support to a pregnant partner or accompanied her to an ANC visit were often mocked by friends [7].

A systematic review of male involvement in prevention of mother-to-child transmission interventions in sub-Saharan Africa [8] found that men who visited health facilities for HIV testing during ANC visits often felt that services were unfriendly to men [9–12] felt ignored by providers [10], or felt the facilities were dominated by women as both patients and providers [9]. Audet et al. also found that stigma surrounding HIV was a significant barrier to uptake of ANC [7]. Because HIV testing and counseling is a routine feature of ANC, a woman is often first diagnosed during an ANC visit, leading to the belief in the community that a woman’s uptake of ANC services, particularly if supported by a male partner, may mean that she or he is HIV positive [7].

Since the mid-1990s, there has been a growing recognition of the importance of male engagement in reproductive, maternal, neonatal, child, and adolescent health (RMNCAH) interventions [13–15]. The World Health Organization (WHO) now includes it as a critical component of ANC to improve birth outcomes [16]. A systematic review by Tokhi et al. of the effectiveness of interventions to involve male partners in improving maternal and newborn health found that men’s involvement improved ANC attendance, institutional delivery, postpartum care, and maternal nutrition [15]. Evidence was less clear on the impact of male engagement interventions on their participation in birth preparedness and complication readiness (BPCR), with some studies showing an effect, and others not [15].

BPCR is a counseling approach that includes elements of antenatal, intrapartum, postpartum, and neonatal care. While the WHO defines BPCR more expansively with as many as nine elements [17], BPCR typically involves learning about danger signs that indicate complications, choosing a health facility for care and delivery, saving money, arranging transport, and choosing a birth companion [17]. BPCR is known to be a powerful intervention to promote the use of skilled care at birth and timely use of facility-based care for obstetric and newborn complications [16, 17].

Tokhi et al. [15] also found that men can provide “substantial practical, financial and emotional support to women and children to overcome demand-side barriers to accessing health services.” Men’s support can normalize care-seeking, improve healthy behavior; increase couples’ communication, and promote equitable decision-making [15]. There is substantial literature showing that couples’ communication about family planning (FP) is positively associated with not only modern contraceptive use [18] but also a range of other health behaviors such as ANC attendance, institutional deliveries, and getting an HIV test during ANC [19] Interventional studies in South Africa and India found that facility-based education for men and women increased couples’ communication about breastfeeding in both countries, the health of their baby in India, and child immunization in South Africa [16].

The State of the World’s Fathers 2015 report [20] found that engaging men early—in ways that women want in ANC, childbirth, and postnatal care— can improve women’s use of maternal and newborn health services, as well as fathers’ long-term support and involvement in the lives of their children. Research from low- and middle-income countries found that male involvement is significantly associated with improved rates of skilled birth attendance and utilization of postnatal care. In high-income countries, the presence of fathers has been shown to encourage and support mothers to breastfeed. Fathers’ support also influences women’s decision to immunize their children and to seek care for childhood illnesses [20].

A randomized controlled trial evaluating the impact of a couples-focused, community-based intervention in Rwanda found that “culturally adapted gender-transformative interventions with men and couples can be effective at changing deeply entrenched gender inequalities and a range of health-related behavioral outcomes.” Participants in the intervention had significant increases in modern contraceptive use, sharing responsibility for housework and childcare, inclusion of women in household financial decision-making, and a modest impact on increased ANC visits. Participants also had reductions in physical and sexual violence against women, and less physical punishment of children [21].

### Logic framework for male involvement

**Fig. 1:**
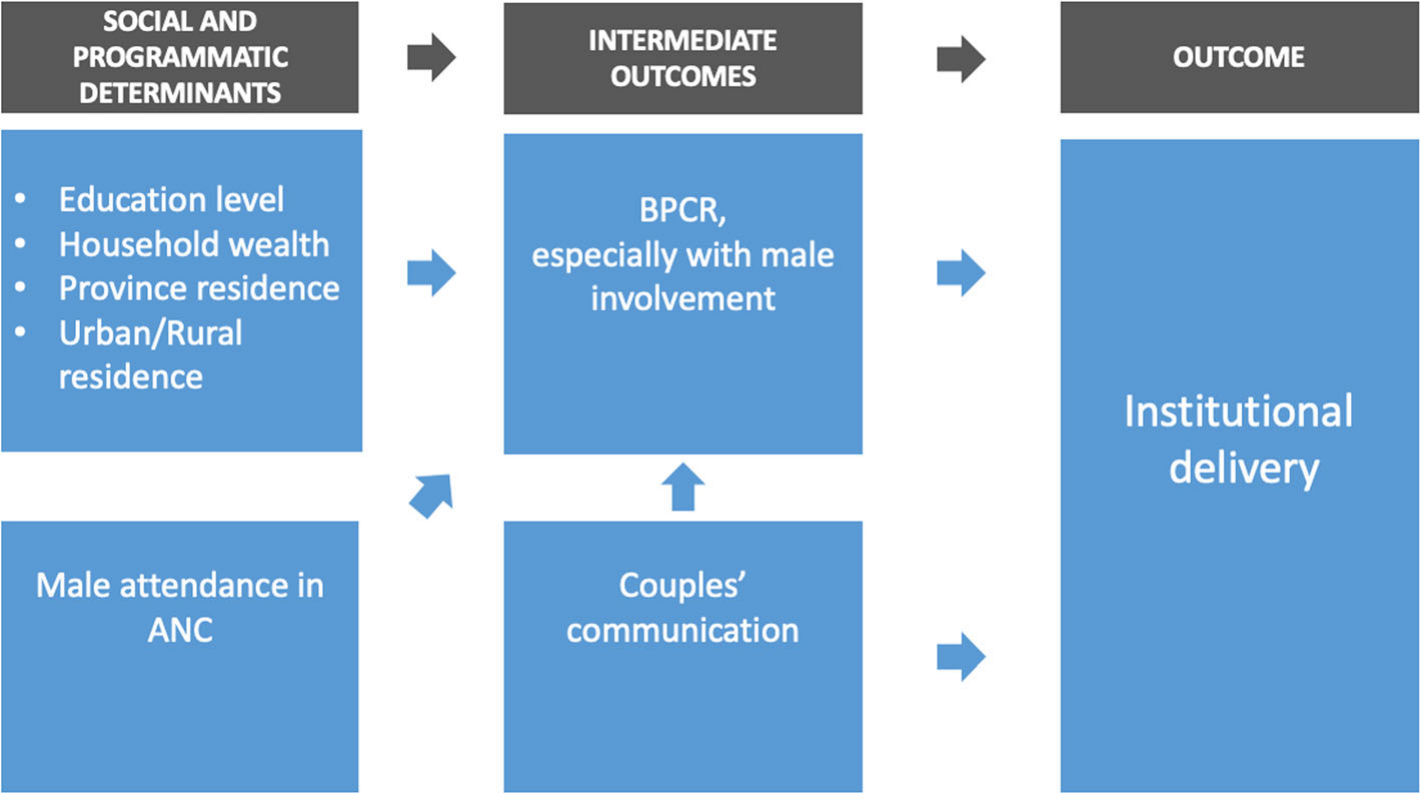
Logic framework

Based on the empirical evidence outlined above and Tokhi et al.’s conceptual framework [15], we propose the logic framework shown in Fig. 1, which considers the specific outcome of institutional delivery. This model explores the impact of sociodemographic factors and illustrates the hypothesis that couple-focused male involvement interventions in RMNCAH, either at the community or facility level, will lead to increased couples’ communication about RMNCAH, increased BPCR, and increased male participation in BPCR. Ultimately, these factors lead to higher rates of institutional delivery.

Our study explores the effects of couples’ communication and male participation in BPCR on institutional delivery. It also explores the interaction of household wealth with these factors.

## Methods

### Study setting and design

The study data came from a baseline, quantitative, cross-sectional household survey conducted in November 2016 with male and female respondents for an integrated maternal and child health project. The survey was conducted in nine districts in Nampula Province (Ribaué, Mecuburi, Eráti, Mossuril, Meconta, Angoche, Moma, Monapo and Nacala Porto) and four districts in Sofala Province (Beira, Dondo, Buzi and Nhamatanda).

### Tool description and implementation

The Knowledge, Practices and Coverage (KPC) survey tool was used for the baseline survey [22]. The survey is divided into 10 modules focused on RMNCAH, as well as modules on demographics, assets, and gender issues. The standard KPC survey instrument was translated into Portuguese, Macua, and Sena. During the five-day interviewer training, the tool was pre-tested in a comparable community in each province that was just outside the project area. In each household, the mother of a 0–23-month-old child was surveyed, asked about her last pregnancy, her own health, and the health of her child. We limited respondents to mothers of 0–23 month-olds because the planned project for which this baseline was conducted was only to last 24 months and we wanted to limit respondents in the endline survey to those who received interventions. Female respondents were interviewed by female data collectors on tablet computers, with the survey loaded in CSPro [23]. Data was sent daily from the field to a central server for data review and tabulation.

### Sampling methods and study subjects

The Mozambique National Statistical Institute (INE) conducted data collection using the same two-stage stratified cluster sampling methodology they used for the 2015 IMASIDA [2]. In the first stage, INE used the latest national census, done in 2007, as the sampling frame. The enumeration areas (EAs) were arranged geographically by administrative area, locality, and village (or by neighborhood, in the case of urban EAs). INE chose EAs from this ordered list in a systematic random fashion, with probability proportional to population size. In the second sampling stage, an onsite supervisor who had a map of the EA did a line listing of households, identifying all households with eligible respondents. These were households with women 15 years or older with a child 0–23 months of age (age ≥ 18 with her consent; age 15–17 with the additional consent of one of her parents). For the purposes of this study, only heterosexual couples were considered. We did include a men’s module in which some of the same questions were asked, and found that men were more likely to report they were involved than their female partner. We decided to use the women’s responses, given the likely stronger social desirability bias on the part of men. After a random start, eligible households were systematically sampled until 15 households were visited. Since the geographic area under study is homogenous, there was clumping of wealth scores towards the bottom half of the distribution. Hence the households were divided into three categories consisting of the lowest 60%, less poor 20%, and highest 20%.

### Sample size

The sample size was calculated according to the needs of the integrated maternal and child health project, which used this survey as a baseline, with planned comparisons at endline. We used an estimated design effect of 1.7, which is standard for KPC cluster surveys of this type. There was a desire to show baseline-to-endline differences of at least 10% for multiple variables of interest, including those starting from a 50% baseline value, with 80% power and a 0.05 level of significance. A 10% non-response rate was assumed. Using the standard formula for a Z statistic for comparison of proportions, this gave a sample of 720 women in the project area in each province and a total sample of 1440 in both provinces, since the sampling was designed to give representative samples in the project area in each province. Fifteen households were selected in 48 clusters in each province (i.e., 96 total clusters).

A post hoc power calculation was performed using a 0.05 level of significance and 80% power, with various levels of intraclass correlation coefficients (ICC) and reference values for variables of interest in the data set. For the outcome of institutional delivery, which had a baseline indicator value of 84% and ICC of 0.17, the minimum detectable difference between two groups would have been 11%.

### Analysis

A wealth index was calculated using the list of household assets according to the methodology adopted by the Demographic and Health Survey (DHS). Using principal components analysis, scores were generated for rural and urban households separately and combined into a single score. Due to the distribution of the scores, the households were divided into three categories consisting of the lowest 60%, less poor 20%, and highest 20% [24].

The independent variables examined were age (15–24, 25–39, 40–49); education level (no schooling, primary, or secondary and higher); province of residence (Nampula or Sofala); place of residence (urban or rural); wealth categories (lowest 60%, less poor 20%, highest 20%); and male participation in ANC. Because the study took place in only two provinces with relatively homogenous populations within each province, we did not examine religion or ethnicity.

The woman’s report of discussing an FP method with partner was used as a proxy for the status of the couples’ communication practices about reproductive health. This was defined as a woman reporting that she discussed the use of an FP method with her partner in the last 12 months. Institutional delivery was defined as a childbirth occurring at a health facility. We chose institutional delivery rather than the presence of a skilled birth attendant because the overwhelming majority of deliveries in Mozambican health facilities are overseen by skilled birth attendants [1], and women often have difficulty accurately reporting distinct cadres of healthcare professionals as “skilled” or “unskilled” compared to the physical location where they delivered [25]. Women were asked separately about each of four elements of BPCR that are germane to male participation and couples’ communication: saving money, arranging transport, choosing a birth companion, and choosing a delivery site [26]. Women were also asked separately about their partners’ participation in each of the four elements of BPCR. Hybrid variables were created for each element of BPCR, assigning a value of zero to women who did not report conducting that BPCR element, “1” to women who carried out the BPCR element without their partners’ participation, and “2” to women who did so with the help of their male partners. The results for BPCR are shown as the sum of the number of elements carried out, as well as participation in each of the elements separately, either with or without male partner involvement.

**Fig. 2:**
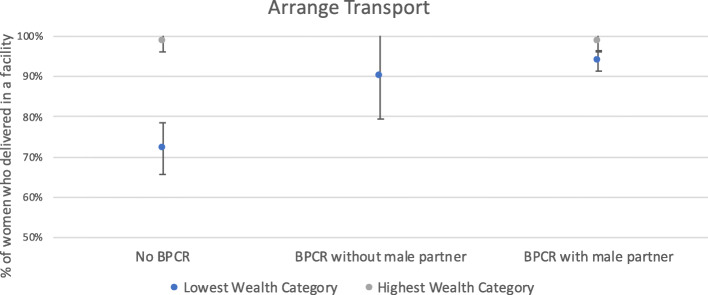
Association between arranging transport and institutional delivery, by wealth category

Data were analyzed in STATA version 14.2 [27]. Frequencies and percentages are used to present descriptive statistics. Multivariable logistic regressions were done to test the association between the variables shown in Fig. 2. Bivariate analysis was performed between the variables used as outcomes in the multivariable models and the independent variables described above. The results are not presented in the manuscript but were used in the selection of control variables in the multivariable model. The effective sample size was smaller than the total number of surveys for some variables included in the models because of responses of “Don’t Know,” which were ignored. The clustering due to the survey design at the level of the enumeration area was accounted for by the use of Huber-White Sandwich estimators. Confidence intervals (CIs) at the 95% level are presented after adjustment for the independent variables listed above.

### Ethical considerations

This study received ethical approval from the National Bioethics Committee of Mozambique and the Johns Hopkins Bloomberg School of Public Health Institutional Review Board (IRB).

## Results

### Description of sample

A total of 1422 women were interviewed out of a total sample of 1440, representing a 99% response rate. Even though these data are from a baseline survey for a project in only two of Mozambique’s 11 provinces, they draw from a sample that is fairly typical as a cross-section of the overall population of Mozambique. Table 1 describes characteristics of the sample according to four demographic variables: province of residence (a proxy for cultural differences between Sofala in the south, mainly Christian and animist, and Nampula in the north, heavily Muslim and matrilineal), maternal age, educational attainment, and urban/rural residence. About 34% of the women in the sample had not completed any schooling, compared to 26% of women in the country as a whole [2]. Seventy-six percent of the sample lived in rural areas, which is similar to the 68% of the national population living in rural areas in 2016 [28].

**Table 1:** Sample characteristics

	Total (*N* = 1422)	
	n	%
**Province**		
Nampula	720	50.6%
Sofala	702	49.4%
**Age**		
15–24	746	52.5%
25–39	606	42.6%
40–49	27	1.9%
Don’t know/No response	43	3.0%
**Education**		
No school	478	33.6%
Primary school	656	46.1%
Secondary school or superior	288	20.3%
**Residence**		
Rural	1077	75.7%
Urban	345	24.3%

### Couples’ communication and utilization of RMNCAH services

Table 2 describes couples’ communication about FP, women’s utilization of ANC and delivery services, and men’s attendance at ANC visits. Over 90% of women attended at least one ANC visit, while 57% of women attended four or more ANC visits. This is similar to ANC coverage rates for Mozambique as a whole, with 93% women reporting at least one ANC visit and 54.6% reporting four or more ANC visits nationally [2]. We found that 84% of women delivered in a health facility, which is higher than the overall national rate of 70% in 2015 [2]. Almost 50% of the women who had attended ANC reported that their partner attended at least one ANC visit with them, which is very similar to the nation as a whole, where the figure from National Health Information System Data was 48% in 2017 [29]. In the table below, only the positive responses are included for brevity.

**Table 2:** Reported couples’ communication and service utilization

	Total (N = 1422)	
	n	%
Couples’ communication on FP in past 12 months		
Yes	501	35.2%
ANC visits during most recent delivery		
At least one visit	1333	93.7%
Four or more visits	807	56.8%
Place of delivery		
Facility	1192	83.8%
Male participation in ANC visit^a^		
Yes	662	49.6%

^a^*N = 1333 for “male participation in ANC”*

### The determinants of BPCR actions with and without male partner involvement

Table 3 provides descriptive information about the number and percentage of women who conducted BPCR, either with or without their male partner. Of the four elements of birth preparedness planning, saving money was the most common, with close to 70% of the women reporting that their partner was involved in saving money with them. Fewer than 60% of women reported that their male partners were involved during their last pregnancy in any of the other three BPCR actions examined: arranging transport, deciding on a birth companion, or choosing the delivery site.

**Table 3:** Birth Preparedness and Complication Readiness

	Total (*N* = 1422)	
	n	%
BPCR		
No action	253	17.8%
Saved money^a^	964	68.2%
Arranged transport	696	48.9%
Chose companion	776	54.6%
Chose delivery site	830	58.4%
All four actions	470	33.1%
All four actions with partner	409	28.8%
BPCR element A: Saved money^a^		
Did not save money	450	31.6%
Saved money without partner	124	8.7%
Saved money with partner	840	59.1%
BPCR element B: Arranged transport		
Did not arrange transport	726	51.1%
Arranged without partner	62	4.4%
Arranged with partner	634	44.6%
BPCR element C: Chose birth companion		
Did not choose companion	646	45.4%
Chose companion without partner	150	10.5%
Chose companion with partner	626	44.0%
BPCR element D: Chose delivery site		
Did not choose delivery site	592	41.6%
Chose delivery site without partner	165	11.6%
Chose delivery site with partner	665	46.8%

^a^*N = 1414 for “saved money”*

### Factors associated with BPCR

Table 4 explores the factors that influence whether or not BPCR was conducted by women, either alone or with their partner. The N in Table 4 is smaller than the overall N of the survey because respondents who had missing values for any of the six variables included in the regression were excluded. Education, province, residence and wealth status, and couples’ communication on FP significantly predicted whether or not BPCR was conducted. Living in Nampula, higher wealth status, male attendance in ANC, and joint communication on FP significantly increased the odds of male participation in BPCR. In fact, the odds of conducting BPCR were 34% higher (aOR 1.34, 95% CI 1.05–1.71, *p* = 0.02) amongst women who communicated with their partner about FP, and the odds were twice as great that they would conduct BPCR *with their male partner* if they communicated with their male partner about FP (aOR 2.09, 95% CI 1.64–2.67, *p* < 0.01)*.* Similarly, the odds of conducting BPCR were 31% higher (aOR 1.31, 95% CI 0.95–1.82, *p* = 0.1) amongst women whose partner attended ANC, and the odds were 54% greater that they would conduct BPCR *with their male partner* (aOR 1.54, 95% CI 1.15–2.07, p < 0.01)*.* Educational status was also associated with increased odds, but not in a simple manner. Those with primary school education reported the highest levels of BPCR and male participation in BPCR, compared to both those with no schooling and secondary or higher education.

**Table 4:** Factors associated with BPCR^a^ and male participation in BPCR^a^

	BPCR (*n* = 1320)				Male participation in BPCR (n = 1320)			
	Adjusted Odds Ratio	95% CI		*p*-value	Adjusted Odds Ratio	95% CI		*p*-value
		min	max			min	max	
Education								
No school	1	1	1	.	1	1	1	.
Primary school	1.54	1.17	2.01	< 0.01*	1.36	1.01	1.84	0.05*
Secondary school or superior	1.37	0.88	2.13	0.16	1.27	0.74	2.18	0.39
Province								
Sofala	1	1	1	.	1	1	1	.
Nampula	3.13	1.96	4.76	< 0.01*	1.79	1.15	2.78	0.01*
Residence								
Rural	1	1	1	.	1	1	1	.
Urban	2.02	1.24	3.29	< 0.01*	1.19	0.72	1.98	0.5
Wealth								
Lowest	1	1	1	.	1	1	1	.
Less poor	1.11	0.81	1.48	0.54	1.09	0.80	1.47	0.59
Richest	2.39	1.48	3.84	< 0.01*	1.85	1.20	2.86	0.01*
Male attendance in ANC								
No	1	1	1	.	1	1	1	.
Yes	1.31	0.95	1.82	0.1	1.54	1.15	2.07	< 0.01*
Couples’ communication on FP								
No	1	1	1	.	1	1	1	.
Yes	1.34	1.05	1.71	0.02*	2.09	1.64	2.67	< 0.01*

* *P*-value significant at 0.05 level

^a^The BPCR indicator was analyzed as an ordinal variable that can take values of 0,1,2,3,4 elements conducted

### The full model—the effect of male ANC attendance, couples’ communication, and BPCR on institutional delivery, controlling for demographic characteristics

Table 5 describes factors associated with institutional delivery. As expected, we found that higher educational attainment and wealth status were strong predictors for institutional delivery. In addition, the odds of delivering in a health facility were 46% higher (aOR 1.46, 95% CI 1.02–2.10, *p* = 0.04) among women who did not do BPCR but reported communicating with their partners about FP than women who did not communicate with their partners about FP. Similarly, odds of delivering in a health facility were 33% greater amongst women who reported communicating about FP with their partner and did BPCR, although the reduced aOR was not found to be significant (aOR 1.33, 95% CI 0.94–1.89, *p* = 0.11).

**Table 5:** Factors associated with institutional delivery

	Institutional delivery without BPCR (*n* = 1320)				Institutional delivery with BPCR (*n* = 1316)			
	Adjusted^a^ Odds Ratio	95% CI		*p*-value	Adjusted^a^ Odds Ratio	95% CI		*p*-value
		min	max			min	max	
Male attendance in ANC								
No	1	1	1	.	1	1	1	.
Yes	1.22	0.81	1.83	0.35	0.95	0.63	1.43	0.81
Couples’ communication on FP								
No	1	1	1	.	1	1	1	.
Yes	1.46	1.02	2.10	0.04*	1.33	0.94	1.89	0.11
BPCR element A: Save money								
No BPCR	.	.	.	.	1	1	1	.
BPCR without partner	.	.	.	.	0.71	0.38	1.30	0.27
BPCR with partner	.	.	.	.	1.12	0.73	1.73	0.60
BPCR element B: Arrange transport								
No BPCR	.	.	.	.	1	1	1	.
BPCR without partner	.	.	.	.	3.88	1.03	14.62	0.05*
BPCR with partner	.	.	.	.	4.31	2.64	7.02	< 0.01*
BPCR element C: Choose birth companion								
No BPCR	.	.	.	.	1	1	1	.
BPCR without partner	.	.	.	.	0.70	0.39	1.25	0.22
BPCR with partner	.	.	.	.	0.76	0.51	1.15	0.20
BPCR element D: Choose delivery site								
No BPCR	.	.	.	.	1	1	1	.
BPCR without partner	.	.	.	.	1.52	0.81	2.83	0.19
BPCR with partner	.	.	.	.	1.98	1.16	3.36	0.01*

* *P*-value significant at 0.05 level. ^a^Adjusted for education, province, residence, wealth

After controlling for education, province, residence, wealth, male attendance in ANC, and couples’ communication, there is a significant correlation between the BPCR element of a woman arranging transport on her own and giving birth in a facility (aOR 3.88, 95% CI 1.03–14.62, *p* = 0.05), but when done *with her partner*, this association is even stronger (aOR 4.31, 95% CI 2.64–7.02, *p* < 0.01). Furthermore, while a woman choosing a delivery site on her own had an association with institutional delivery which did not reach statistical significance (aOR 1.52, 95% CI 0.81–2.83, *p* = 0.19), the odds of delivering in a facility were nearly twice as great amongst women whose partners helped to choose a delivery site (aOR 1.98, 95% CI 1.16–3.36, *p* = 0.01).

### Male involvement in BPCR is associated with a narrowed gap in institutional delivery rates between wealth categories

**Fig. 3:**
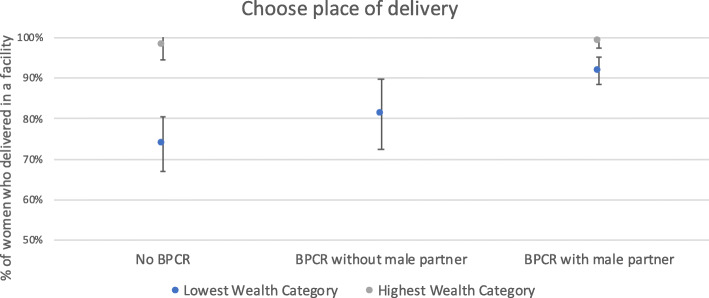
Association between deciding on place of delivery and institutional delivery, by wealth category

Figures 2 and 3 show the associations between elements of BPCR that were significant (arranging transport and choosing a delivery site) and institutional delivery rates by the highest and lowest wealth categories. Note that all women in the highest wealth category arranged transport or chose a place of delivery with their male partner. Among women who did not arrange transport (no BPCR), 99% of those in the highest wealth category had an institutional delivery, compared to only 72% of women in the lowest wealth category. Among those who arranged transport *with their male partners* (BPCR with partner), the difference narrowed to 99% in the highest wealth category and 94% in the lowest delivering in a facility.

A similar pattern is seen among women who chose the delivery location. Among women who did not choose a delivery location (No BPCR), 98% of women in the highest wealth category had an institutional delivery, compared to only 74% of women in the lowest wealth category. Among women who did choose a delivery location with their partners (BPCR with partner), 98% of women in the highest and 92% of women in the lowest wealth category had an institutional delivery.

## Discussion

Our findings contribute to the increasing global evidence-base that men play an important role in facilitating positive maternal and newborn health behaviors, leading to better outcomes. Increased communication and joint decision-making with partners can lead to increased institutional delivery. The recent systematic review on the effectiveness of interventions to increase involvement of men to improve maternal and newborn health by Tokhi et al. [15] included several interventional studies with positive results on institutional delivery. The authors also found positive effects on couples’ communication around a number of health practices, including FP in India [30], breastfeeding in India, South Africa, and Turkey [30–32], and child immunization South Africa [31]. However, the interventional studies that specifically looked at the effect of male involvement on BPCR showed varying results.

Our study found that women whose partners helped them arrange transport or choose a place of delivery were even more likely to have an institutional delivery than the women who carried out these tasks alone. It was already well-established that BPCR is causatively associated with institutional delivery [17]. What is striking in this study is that the odds ratio increased for both arranging transport and choosing a place of delivery when partners were involved in the decision, compared to when women made the decision on their own.

Furthermore, our findings indicate that couples communicated about FP was associated with increased BPCR and rates of institutional delivery. This suggests that the effect of couples’ communication on institutional delivery is only partially mediated through the BPCR elements measured. In other words, couples’ communication is about more than FP and BPCR and is positively and independently associated with the outcome of institutional delivery. A recent trial in Rwanda found that promoting gender transformative dialogue and couple communication can improve a range of health behaviors [1].

One finding appears to be inconsistent, that is, arranging transport was found to be associated with institutional delivery, but saving money was not. We assume that the money saved was most often used for transport, since there is no cost associated with pregnancy or delivery in a public facility in Mozambique. This warrants further research.

Male participation in ANC had a significant association with male participation in BPCR, and this may be because BPCR is encouraged by providers during ANC visits. Although we cannot ascertain causality due to the cross-sectional nature of this study, the results suggest that male participation in ANC led to their increased knowledge about the importance of BPCR for a healthy and safe delivery. Other studies have found that interventions to increase men’s education about maternal and newborn health have led to increased BPCR [33–35]; however, we do not know to what extent men’s knowledge of maternal and newborn health increased as a result of their participation in ANC.

Our findings are consistent with the logic framework presented here, based on the theory of change proposed by Tokhi et al. [15]. Our framework posits that improved couples’ communication is a step towards improving male support to women in accessing RMNCAH services. It is worth noting that we found that not all the effects of couples’ communication could be explained through BPCR. There was some residual, unexplained association between couples’ communication and institutional delivery. Possible explanations are that the elements of BPCR measured in the survey do not fully encompass the critical aspects of BPCR (e.g., finding someone to care of children); that communication operates through another mechanism, such as immediate joint problem-solving as opposed to planning beforehand for the birth; or that communication is a proxy for an even larger set of behaviors and aspects of the relationship. These possibilities need to be explored through further research, particularly qualitative studies.

Although we do not know if the couples’ communication in this study took place before or after birth, there is increasing evidence that men’s knowledge [35] and couples’ communication about FP are positively associated with modern contraceptive use [18] and a range of other health behaviors, such as attendance to ANC, institutional deliveries, and getting an HIV test during ANC [19, 36–38]. Community-based interventions, particularly with young married couples, have also demonstrated increased contraceptive use, use of ANC, and birth preparedness. A systematic review of such interventions concluded that, “community based interventions targeting young married couples, their immediate family, community members and health service providers contribute positively to improving access and utilization of reproductive health services in resource-constrained settings of low and middle-income countries” [39]. Moreover, it is interesting to note that the odds ratio for couples’ communication did not change when adjusted for wealth, education, or place of residence. This finding is consistent with the idea that couples’ communication and joint decision-making can be equally effective across demographic categories. A 2018 report by the United States Agency for International Development on involving men and boys to improve FP outcomes looked at DHS data from 40 countries on men’s attitudes about reproductive health. The report concluded that men who have more equitable attitudes towards FP (e.g. such as believing it is both the man’s and the woman’s responsibility) and about family roles (e.g., men who share decision-making with women) are more likely to report using an FP method. The report also found that open communication about FP between couples helps facilitate men’s support for—and women’s use of—contraception [40].

Another important finding from our study was that male participation seems to narrow the gap between the lowest and highest wealth categories in terms of institutional delivery. Nearly all women in the highest category had an institutional delivery. In comparison, 72% of women in the lowest category who did not arrange transport had an institutional delivery. But 90% of poor women who arranged transport alone delivered in a facility as did 94% of poor women who arranged transport with their male partner. A similar pattern was seen among poor women with respect to choosing a birth location: 74% who did not choose a location had a facility delivery, as did 81% who chose a location without their partner and 92% of those who chose with their partner. These findings confirm a growing body of research that demonstrates the importance of addressing the intersectionality of gender inequality with other constraints and vulnerabilities, such as poverty and other social stigmas, when accessing maternal newborn care [41, 42]. There may be a number of reasons why men’s participation in BRCP narrows the gap of institutional delivery between the rich and the poor. Men’s participation may, for example, make up for the fact that women in the poorer group may not be able to afford transport on their own but can do so with the help of their male partners. These should be explored through further research, including qualitative means.

Because this was a cross-sectional survey, it is not possible to draw conclusions regarding the way in which decisions were made about BPCR or, ultimately, institutional deliveries. It is possible that male participation, without intentional efforts to promote gender equity in couples’ communication and decision-making, could lead to the erosion of women’s agency in decision-making. For example, the qualitative study by Audet, et al. on the barriers to male involvement in ANC in rural Mozambique found that men’s desire or perceived right to control their pregnant partners may be the root of some men’s interest to be involved in ANC [7]. The WHO recognizes that male involvement is only helpful when it is “implemented in a way that respects, promotes and facilitates women’s choices and their autonomy in decision-making and supports women in taking care of themselves and their newborns” [16]. In some cases, male involvement could be an aspect of control, i.e. a lack of gender equity, and in others, an expression of egalitarian concern and care. Similarly, a woman’s ability to plan and execute BPCR on her own could be seen as an expression of her own autonomy or as a strategy used by women in otherwise highly inequitable situations. This is an area where additional qualitative research would be helpful. Others have found that in African countries, gender equality, as measured by women’s level of decision-making regarding health care, the purchase of major household goods, the purchase of daily goods, and visits to family/friends, as well as attitudes opposing gender-based violence, is associated with institutional deliveries, child immunization, and care-seeking for child health [43]. On the other hand, according to the DHS, men in Africa are still key decision makers in health overall, and, therefore, their support for institutional delivery would logically increase its likelihood However, there may be other underlying factors causing the association, which deserve further exploration.

### Strengths and limitations

Despite being a small study in two provinces, this study yielded important information about the association between couples’ communication, male engagement, and improved health behaviors. This poses important implications for policy makers and program managers when considering approaches and populations to include in reproductive and maternal health programming.

The greatest limitation is that the study was cross-sectional and not interventional; therefore, the extent to which correlations are causative is only suggestive. For instance, we did not ask if the current partner was the same partner who impregnated the woman, nor if the communication on FP preceded the birth or came afterwards. We also did not measure the distance from respondent’s communities to the nearest health facility, a factor that is known to have an effect on institutional delivery rates. The study relied on self-reporting, and could have been affected by recall bias and social desirability bias, for example, when women were asked about their male partner’s behavior.

An additional limitation is that the data for this analysis came from a household survey that served as the baseline for a project intervening in multiple RMNCAH areas (maternal health, nutrition, immunization, etc.), but not specifically designed for the purposes of evaluating male engagement and couples’ communication. This means that we were not able to ask additional in-depth questions because the survey already took almost 2 h to administer. The sample size calculation for the survey did not take into account the likelihood of small cell sizes for some sub-analyses we performed, such as the interaction of wealth and BPCR. Given the large number of technical areas that the baseline study needed to cover, it was not feasible to delve into any issue in great detail in the survey instrument. In terms of measuring couples’ communication, the one question that was included was specifically about communication about FP, but there were no questions about communication on other RMNCAH topics. We have, however, presented some published evidence that couples’ communication on FP has previously been associated with other RMNCAH outcomes when used as a proxy for more general communication on RMNCAH. We were not able to explore issues of either the quality of male engagement, i.e., the extent to which it might be coercive, or women’s empowerment; e.g., did male engagement decrease female agency or interact with it in significant ways? We plan to explore these issues in a mixed methods endline evaluation of the project’s gender interventions for couples’ communication and male engagement through a repeat of the quantitative KPC survey and a qualitative component. Finally, it was beyond the scope of this paper to explore the nature of cultural differences between provinces and their exact relation to male participation for birth preparation and in the birthing process itself. However, we tried to take account of these differences by including “province” as a dummy variable in all regression models that we ran.

## Conclusion

Couples’ communication matters. Strategies to promote couples’ communication in RMNCAH interventions should be encouraged by MOH and policies and guidelines for both facility-based health provider practices and community-based health promotion efforts. Couples’ communication’s effect on institutional delivery appears to only be partially mediated through BPCR. The ways in which couples’ communication contributes to BPCR and to institutional delivery, whether it represents communication beyond BPCR, or whether other factors in the relationship are at play, still need further exploration through qualitative means. More evidence is also needed on the effectiveness of couple communication across a variety of settings. There are two main components of BPCR we found to be associated with higher institutional delivery rates—arranging transport and choosing location of delivery—and male engagement strengthens their association. BPCR, especially BPCR with male involvement, seems to close the gap between the wealth categories in terms of institutional delivery rates. This suggests that male engagement and couples’ communication interventions could be an important intervention for improving RMNCAH among women in lower wealth categories. Further qualitative studies need to be done to explore these findings in depth.

## Data Availability

The data that support these findings are available upon reasonable request to the corresponding author.

## Abbreviations

ANC: Antenatal care
aOR: Adjusted odds ratio
BPCR: Birth preparedness and complication readiness
CI: Confidence interval
DHS: Demographic and Health Survey
EA: Enumeration areas
FP: Family planning
HIV: Human immunodeficiency virus
ICC: Intraclass correlation coefficient
IMASIDA: National Survey on Immunization, Malaria, and HIV/AIDS
INE: Mozambique National Statistical Institute
IRB: Institutional Review Board
KPC: Knowledge, Practices, and Coverage Survey Tool
MNH: Maternal and newborn health
RMNCAH: Reproductive, maternal, newborn, child and adolescent health
USAID: United States Agency for International Development
WHO: World Health Organization
